# Perceived stigma and barriers to care in UK Armed Forces personnel and veterans with and without probable mental disorders

**DOI:** 10.1186/s40359-019-0351-7

**Published:** 2019-11-27

**Authors:** Victoria Williamson, Neil Greenberg, Sharon A. M. Stevelink

**Affiliations:** 1 King’s Centre for Military Health Research, Institute of Psychology, Psychiatry and Neuroscience, King’s College London, 10 Cutcombe Road, London, SE5 9RJ UK; 2 Department of Psychological Medicine, Institute of Psychology, Psychiatry and Neuroscience, King’s College London, 16 DeCrespigny Park, London, SE5 8AF UK

**Keywords:** Stigma, Barriers to care, Attitudes, Military personnel, Veterans, Mental health, Post-traumatic stress disorder, Common mental disorders, Alcohol misuse

## Abstract

**Background:**

Previous studies have found that perceptions of mental health related stigma can negatively impact help-seeking, particularly in military samples. Moreover, perceptions of stigma and barriers to care can vary between individuals with different psychiatric disorders. The aim of this study was to examine whether perceptions of stigma and barriers to care differed in a UK military sample between those with and without a current likely mental health diagnosis.

**Method:**

Structured telephone interviews were carried out with 1432 service personnel and veterans who reported recent subjective mental ill health in the last 3 years. Participants completed self-reported measures relating to perceived stigma, barriers to care and psychological wellbeing.

**Results:**

Those meeting criteria for probable common mental disorders (CMD) and PTSD were significantly more likely to report concerns relating to perceived and internalised stigma and barriers to care compared to participants without a likely mental disorder. Compared to individuals with likely CMD and alcohol misuse, those with probable PTSD reported higher levels of stigma-related concerns and barriers to care – although this difference was not significantly different.

**Conclusions:**

These results indicate that perceptions of stigma continue to exist in UK serving personnel and military veterans with current probable mental disorders. Efforts to address particular concerns (e.g. being seen as weak; difficulty accessing appointments) may be worthwhile and, ultimately, lead to improvements in military personnel and veteran wellbeing.

## Background

Mental health-related stigma and a lack of trust/confidence in mental health providers has been found to be an important barrier preventing access to necessary psychological care in several high pressure occupations, including healthcare providers, first responders and military personnel [1, 2]. In recent years, the underutilization of mental health services by serving and ex-serving military personnel has received growing research attention [3–5]. In both US and UK military samples, perceptions of mental health stigma, self-reliance and concerns that seeking mental health services may negatively impact one’s military career and have been identified as key reasons for not seeking psychological treatment [6]. Several types of stigma exist which are thought to interact and contribute towards barriers to help-seeking [7]. Common types of perceived stigma include public stigma, internalised stigma and structural discrimination. Public stigma, where others endorse prejudice against those with mental health problems [8], in an Armed Forces (AF) context can relate to concerns of being perceived as less competent by colleagues or experiencing differential treatment by unit leaders. On the other hand, internalised stigma occurs when an individual with mental health difficulties internalises negative stereotypes held by society resulting in poorer self-esteem and feelings of shame [9, 10]. In an AF context, commonly described internalised stigma beliefs include ‘I am weak’ and ‘I am crazy’ [7]. Finally, structural discrimination can be experienced when an organisation’s regulations may (un)intentionally disadvantage those with mental health difficulties [11]. Examples of structural discrimination can include perceived difficulty taking time off work for an appointment or a lack of resources to access help [7].

The recent research studies focusing on mental health and stigma have contributed towards the promotion of mental health-related discussions, with various campaigns run within the military (i.e. “don’t bottle it up”) and general public (i.e. “no health without mental health”, “mental health matters”) in an effort to reduce mental health stigma and encourage help-seeking [12]. There is some evidence that stigma-related beliefs can vary between individuals with different psychiatric disorders. For example, Iversen et al. [5] found serving and ex-serving personnel with post-traumatic stress disorder (PTSD) were more likely to report more internalised stigma and barriers to psychological care compared to individuals with non-PTSD mental health problems and those without a diagnosis of a mental disorder [5]. An examination of whether mental health-related stigma perceptions continue to vary by mental disorder may inform future interventions to more effectively reduce stigma and improve help-seeking. The present study aimed to examine perceptions of stigma and whether rates of perceived stigma, internalised stigma and barriers to care differ in a UK military sample with current probable PTSD, depression, anxiety, or alcohol misuse and those without a likely mental health disorder.

## Methods

Ethical approval was sought and obtained from the UK Ministry of Defence Research Ethics Committee (ref: 535/MODREC/14).

### Participants

The present tri-service sample was based on a large sample of regular, reserve and veteran UK AF personnel who completed the third phase of an existing cohort study [13] Further details regarding the cohort study are described by Fear et al., 2010; Hotopf et al., 2006; and Rona et al., 2006 [14–16]. The sample for the current study was drawn from the pool of individuals in phase three who gave consent to future contact and answered ‘yes’ to the question, ‘have you had a mental health, stress or emotional problem in the past three years?’. Of the 2030 eligible participants, 1713 were selected for inclusion. Of these, 263 did not complete the interview and two interviews were lost during a computer service error. Sixteen participants were ultimately not included in the final analysis as they did not meet inclusion criteria. This resulted in a final sample of 1432 participants (83.6% response rate).

### Procedure

The study procedure is described in-depth by Stevelink et al. [17]. As an overview: information and invitations to participate were sent to participants and consenting participants completed a structured telephone interview with responses entered into a secure electronic study database. Study interviews were audio-recorded with participant consent. The interviews lasted between 17 and 148 min (median = 38.5 min). As a thank you for their time, participants received a cheque for £25 (USD $35) on completion of the interview. Data collection was conducted between February 2015 and December 2016.

### Materials

Items from the Perceived Stigma and Barriers to Care for Psychological Problems – Stigma Subscale (PSBCPP-SS), the Barriers to Access Care Evaluation (BACE) measure, and the Self-stigma of Seeking Psychological Help (SSOSH) scale were used to assess mental health-related stigma and barriers to care [3]. Participants were asked to respond to statements relating to i) access to mental health services (4 items), ii) internalised stigma of mental illness (7 items), and iii) perceived stigma of mental health care/providers (9 items; Additional file 1: Table S1). A 5-point Likert scale was used with responses ranging from 1 (strongly disagree) to 5 (strongly agree). Scores were reverse coded to ensure that a higher total score on each subscale indicated a higher perceived level of barriers to care, perceived stigma and negative attitudes. Responses on the measures of stigma were grouped into ‘agree’ (agree and strongly agree) and ‘disagree’ (disagree and strongly disagree). Responses of ‘neither agree nor disagree’ were excluded from this analysis [5]. Three scales (access to mental health services; internalised stigma of mental illness and perceived stigma of mental health care/providers) were created by summing the items pertaining to each domain to create a summary score (see Additional file 1: Table S1). The scale has been widely used among military personnel with good reliability and validity [3, 18].

To determine probable mental health disorders, the Patient Health Questionnaire (PHQ-9 [19]) was used to assess depression symptoms (cut off score of ≥15), the Generalized Anxiety Disorder scale (GAD-7 [20]) was used to assess anxiety symptoms (cut off score of ≥10), the PTSD checklist for DSM-5 (PCL-5 [21]) was used to assess probable PTSD (cut off score ≥ 38), and the Alcohol Use Disorders Identification Test (AUDIT-C [22]) was used to assess likely alcohol misuse (cut of score of ≥10 to indicate substantial alcohol misuse consistent with previous studies in military samples; e.g. [23]).

### Analyses

Descriptive analyses were conducted to provide an overview of the sample characteristics (Table 1). To compare perceptions of stigma and barriers to care amongst those with and without mental health problems, unadjusted and adjusted ratios as well as 95% confidence intervals are provided in Table 2. Odds ratios were calculated using logistic regression analysis and were adjusted for socio-demographic characteristics (age, sex). As individuals could meet likely criteria for more than one disorder, overlapping 95% confidence intervals (CI) were used to indicate non-significant differences in scores. Common Mental Disorders (CMD) reflects participants who met case criteria on the GAD-7 and/or the PHQ-9. To account for non-response, response weights were generated based on variables shown to be associated with responding (i.e. age, rank and Service). Response weights were calculated as the inverse probability of responding once sampled. To account for weighting, all analyses were performed with survey (svy) commands applied using STATA v.15 (StataCorp, College Station, Texas, USA).

**Table 1:** Participant demographic characteristics

Index	N(%)
Mean age (SD)	41.6 (9.4)
Gender, n (%)	
Male	1213 (84.7%)
Female	219 (15.3%)
Serving status, n (%)	
Serving	783 (54.7%)
Left service	649 (45.3%)
Service branch	
Royal Navy or Royal Marines	197 (13.1%)
Army	937 (65.9%)
Royal Air Force	314 (21.0%)
Rank	
Officer	378 (26.4%)
Non-commissioned officer	867 (60.5%)
Junior rank	187 (13.1%)
Service type, n (%)	
Regular	1167 (81.5%)
Reserve	265 (18.5%)
Met likely diagnostic criteria, n (%)	
PTSD	124 (8.7%)
Alcohol misuse	266 (18.6%)
CMD^a^	276 (19.3%)

*CMD* common mental disorders, includes participants meeting case criteria for likely anxiety and depressive disorders, *PTSD* participants meeting case criteria for posttraumatic stress disorder, *SD* standard deviation

^a^ data missing for 1 participant

**Table 2:** OR and AOR for stigma scale summary scores for individuals meeting case criteria for likely PTSD, alcohol misuse, and common mental disorders versus those who did not meet criteria for each disorder

Stigma scales	PTSD	Alcohol misuse	CMD
Access to mental health services			
OR (95% CI)	**1.81 (1.37; 2.39)**	1.15 (0.91; 1.45)	**1.57 (1.26; 1.97)**
AOR (95% CI)	**1.82 (1.38; 2.40)**	1.17 (0.92; 1.49)	**1.56 (1.26; 1.94)**
Internalised stigma of mental illness			
OR (95% CI)	**1.33 (1.20; 1.46)**	**1.12 (1.04; 1.20)**	**1.20 (1.12; 1.29)**
AOR (95% CI)	**1.33 (1.20; 1.47)**	**1.12 (1.04; 1.21)**	**1.19 (1.11; 1.28)**
Perceived stigma of mental health care/providers			
OR (95% CI)	**1.68 (1.36; 2.08)**	1.15 (0.97; 1.37)	**1.32 (1.12; 1.56)**
AOR (95% CI)	**1.68 (1.36; 2.07)**	1.18 (0.99; 1.40)	**1.30 (1.09; 1.55)**

*CMD* common mental disorders, includes participants meeting case criteria on the GAD and PHQ-9, *OR* odds ratios, *AOR* adjusted odds ratio. Adjusted for sex and age. Results for those who responded ‘neither agree nor disagree’ are not presented. *CI* confidence interval. Bold values denote statistical significance

## Results

### Participant characteristics

Of the 1432 participants, 85% were male (*n* = 1213) and 54.7% were in active service at the time of data collection (*n* = 783; see Table 1). The majority of participants had been in regular service rather than reserve (81.5% vs 18.5%). A moderate proportion of participants met criteria for probable mental health problems on the psychometric measures, with 8.7% meeting criteria for likely PTSD (*n* = 124), 18.6% meeting criteria for alcohol misuse (*n* = 266), and 19.3% meeting criteria for CMD (*n* = 276). It should be noted that these are not prevalence rates or representative of mental disorder prevalence in the AF as participants were included if they self-reported mental health, stress or emotional problems.

### Perceived stigma and barriers to care

Individuals meeting criteria for current probable PTSD and CMD were consistently more likely to endorse internalised stigma of mental illness, perceived stigma of mental health care/providers and difficulties with access to care compared to those who did not meet case criteria (Table 2, Additional file 1: Table S2). Odds ratios found for individuals with probable PTSD were greater than the odds ratios of those meeting criteria for likely CMD or alcohol misuse across all three scales; however, overlapping confidence intervals indicate these differences may not be statistically significant. Although, differences in effects sizes between those meeting probable PTSD criteria and individuals meeting likely alcohol misuse criteria are approaching significance for the internalised stigma of mental illness and perceived stigma of mental health care/provider scales.

The relationship between probable mental disorders and endorsement of the access to mental health services as well as internalised and perceived stigma items is described below.

#### Access to mental health services

In terms of practical barriers to accessing treatment, compared to those who did not meet criteria for a probable mental disorder, those meeting criteria for PTSD were significantly more likely to report access problems (AOR 1.82; 95% CI 1.38; 2.40; Table 2). In particular, individuals meeting case criteria for probable PTSD significantly more commonly endorsed concerns that ‘it would be difficult to schedule an appointment’ (32.5% agreement in cases versus 12.5% non-cases, Additional file 1: Table S1) and it would be difficult to get time off work for treatment compared to those not meeting probable PTSD criteria (29.2% PTSD cases agree/strongly agree vs 15.8% of non-cases).

Access-related concerns were also significantly more commonly reported by those meeting criteria for probable CMD compared to those who did not meet case criteria (AOR 1.56; 95% CI 1.26; 1.94) (Table 2). Similar to PTSD cases, those meeting criteria for probable CMD were more likely to report that ‘it is difficult to schedule an appointment’ (25.9% of CMD cases agree/strongly agree vs 11.6% of non-cases) and ‘it would be difficult to get time off work for treatment’ (26.7% of CMD cases agree/strongly agree vs 14.7% of non-cases) (Additional file 1: Table S1).

Those meeting criteria for probable alcohol misuse were not significantly more likely to report access problems compared to individuals not meeting case criteria (AOR 1.17; 95% CI 0.92; 1.49) (Table 2). Those meeting criteria for probable alcohol misuse did not noticeably endorse any particular access-related items compared to those not meeting criteria.

#### Internalised stigma of mental illness

Internalised mental health-related stigma was significantly more likely to be reported in those meeting criteria for probable PTSD compared to those not meeting criteria (AOR 1.33; 95% CI 1.20, 1.47) (Table 2). A number of items were strongly endorsed by individuals with probable PTSD, including concerns that ‘my unit bosses might treat me differently’ (73.8% PTSD cases agree/strongly agree vs 46.0% of non-cases) and ‘members of my unit might have less confidence in me’ (62.9% PTSD cases agree/strongly agree vs 41.1% of non-cases) (Additional file 1: Table S1).

Compared to those not meeting criteria, individuals meeting criteria for probable CMD were significantly more likely to report concerns relating to the stigmatisation of mental illness (AOR 1.19; 95% CI 1.11, 1.28); including ‘I would be seen as weak by those who are important to me’ (56.9% CMD cases agree/strongly agree vs 36.4% of non-cases) and ‘my boss would blame me for the problem’ (33.6% CMD cases agree/strongly agree vs 13.1% of non-cases).

Individuals meeting criteria for probable alcohol misuse were significantly more likely than those not meeting criteria to report mental health related stigma (AOR 1.12; 95% CI 1.04; 1.21) (Table 2). In particular, ‘concerns about what my friends and family might think’ was a more commonly endorsed item by those meeting probable alcohol misuse case criteria (45.4% cases agree/strongly agree vs 29.9% of non-cases) (Additional file 1: Table S1).

#### Perceived stigma of mental health care/providers

Compared to those not meeting criteria, individuals meeting criteria for probable PTSD were significantly more likely to report issues related to mental health treatment and service providers (AOR 1.68; 95% CI 1.36; 2.07) (Table 2). Items, including ‘mental health care doesn’t work’ (20.1% PTSD cases agree/strongly agree vs 5.9% of non-cases) and ‘my bosses discourage the use of mental health services’ (13.4% PTSD cases agree/strongly agree vs 3.3% of non-cases) were particularly commonly endorsed by those meeting probable case criteria for PTSD (Additional file 1: Table S1).

In comparison to those not meeting criteria, participants meeting probable CMD criteria were also significantly more likely to report concerns relating to mental health treatment and service providers (AOR 1.30; 95% CI 1.09; 1.55) (Table 2). Those with probable CMD strongly endorsed several items, including ‘not wanting a mental health problem to be on my medical records’ (59.3% CMD cases agree/strongly agree vs 44.2% of non-cases) and ‘I’ve had bad experiences with mental health professionals’ (23.5% CMD cases agree/strongly agree vs 10.1% of non-cases) (Additional file 1: Table S1).

Those meeting criteria for probable alcohol misuse were not significantly more likely to report concerns relating mental health treatment and service providers compared to those who did not meet diagnostic criteria (AOR 1.18; 95% CI 0.99; 1.40), although this effect is approaching statistical significance (Table 2). Nonetheless, compared to those who did not meet criteria, individuals meeting criteria for probable alcohol misuse particularly endorsed ‘wanting to solve the problem on my own’ (75.3% cases agree/strongly agree vs 58.9% of non-cases). This high-level of self-reliance was also more frequently endorsed by those meeting probable CMD and PTSD criteria compared to non-cases. Moreover, in comparison to those not meeting criteria, individuals with probable alcohol misuse were more likely to report concerns that ‘my visit would not remain confidential’ (15.4% cases agree/strongly agree vs 9.6% of non-cases) (Additional file 1: Table S1).

## Discussion

This study had three main findings. First, across all items, those meeting criteria for current probable CMD and PTSD were more likely to report concerns relating to access to care, internalised mental health-related stigma and perceived stigma of mental health treatment/service providers compared to those who did not meet case criteria. Second, compared to those not meeting diagnostic criteria, participants with probable alcohol misuse disorders generally did not report higher levels of stigma or barriers to care. Although, individuals meeting caseness for probable alcohol misuse did more strongly endorse a number of items, including wanting to address mental health problems on their own, compared to non-cases. Finally, across all three subscales, the odds ratios of those with probable PTSD were larger compared to those with probable CMD or alcohol misuse, indicating that participants with probable PTSD experience the greatest perceived and internalised stigma and barriers to care; although, overlapping confidence intervals suggest this difference is not statistically significant.

The results of this study indicate that, despite efforts to reduce the stigma of mental illness and encourage discussion in both the military and general population [12], considerable mental health related stigma and barriers to care exist for those with probable mental health problems. This is inconsistent with previous studies showing that improved mental health literacy led to more positive attitudes towards help seeking in the general public [24, 25]. However, our findings may reflect that our sample was comprised of those who self-reported mental health or emotional/stress problem in the last 3 years. Nonetheless, our results are in line with previous studies of mental health-related stigma in military samples [3, 5]. Moreover, our findings indicate that the type and strength of perceptions of stigma and barriers to care may vary between individuals with different psychological problems. For example, those with probable PTSD were found to most strongly endorse items on the stigma scales in the present study. This difference in perceived stigma and help-seeking may reflect variability in the understanding of different mental disorders in the AF community. This continued existence of mental health stigma within the AF community potentially reflects that an omnibus, one-size-fits-all approach for tackling mental health stigma is ineffective [26]. Recent research indicates that improved self-awareness of having a mental health problem and a positive first experience when engaging with psychological treatment may be particularly effective in overcoming barriers to care [6]. It is possible that continuing to raise awareness of mental health problems and symptoms, as well as publicising positive patient testimonies may improve knowledge. Indeed, across the sample, very few participants indicated that they did not know where to go for psychological support. Whilst this is a positive finding, our data show that simply knowing what support exists does not mean that people will access it. To date, few studies have examined the practical impact of recent campaigns to reduce mental health-related stigma (e.g. ‘don’t bottle it up’; [12]). We suggest that more work is needed to understand how to encourage those who know what services exist, to use them. This appears to be especially important for those suffering with PTSD, possibly because avoidance is a core PTSD symptom [27]. Further examination of this topic for the AF community is warranted given recent studies showing that PTSD rates are elevated in military veterans [13].

It is interesting to note that, compared to those without CMD/PTSD/alcohol misuse, individuals meeting probable case criteria were more likely to endorse concerns that their mental health difficulties may mean they are seen as weak by not only colleagues and their superiors, but also by friends and family members. This perception of stigma is consistent with previous research in military personnel and veterans [5]. Friends and family members are often a key source of support for those with mental health problems and have been found to be central to treatment access and adherence. Recent efforts such as the HALO project (Archer M, Harwood H, Stevelink S, Rafferty L, Greenberg N: Community reinforcement and family training and rates of treatment entry: a systematic review, Under review) which aims to improve family members’ recognition of their veteran’s psychological distress, delivery of low-level support and signposting information may be effective at not only improving wellbeing but also in addressing this type of stigma.

Those with probable PTSD and CMD also reported that it would be challenging to get an appointment. This potentially reflects awareness of extensive waiting list times for care within the UK National Health Service (NHS) or the perceived time commitment required for PTSD/CMD treatment (e.g. regular weekly sessions over an extended period). This study was however carried out prior to the introduction of the TILS (transition, intervention and liaison service) [28] which purports to offer veterans an initial appointment within 2 weeks. However, the results of this study highlight the continued need to ensure that veterans who wish to access care know how to do so. Treatment waiting lists, amongst other personal, economic and political factors, must be considered as well as perceptions of stigma in explaining the complex reasons why individuals can be reluctant to seek and engage with mental health services. Moreover, particularly prominent in cases of likely mental disorders was the endorsement of stigma items relating to a desire for self-agency and to address psychological problems themselves. It is possible that promising programs such as the InDEx app, which aims to improve awareness of alcohol consumption and offers suggestions to reduce consumption [29] may be effective. Further emphasis in public campaigns that psychological treatment provides the skills to more effectively manage one’s own wellbeing, similar to campaigns for physical health (e.g. diet, exercise), could also be particularly beneficial.

### Strengths and weaknesses

A strength of the study is the large sample which had a high response rate (84%) and was diverse in the inclusion of both serving and ex-serving personnel. Furthermore, data collection took place independently of the military and data quality should not be affected by participant concerns that their responses will be reported back to the chain of command. Nonetheless, the presence of probable mental disorders was assessed via self-report questionnaire rather than a clinical interview which is considered the gold-standard for mental disorder assessment. Finally, only those who self-reported having experienced a mental health or emotional/stress related problem in the last 3 years were included in the present study; however, this may have led to the exclusion of individuals with mental health problems who did not disclose their difficulties and limits the generalisability of the results.

## Conclusions

The results of this study indicate that perceptions of stigma continue to exist in serving personnel and military veterans with current probable mental disorders. Although other barriers to care-seeking exist, this study suggests stigma may well impair help-seeking, which is especially important for those suffering with PTSD which has avoidance as one of its core symptoms. We suggest that further efforts to address stigmatising particular concerns (e.g. being seen as weak by family and friends; getting an appointment is challenging) may be worthwhile in order to ensure that military personnel and veterans with mental health are able to access appropriate care.

## Supplementary information

**Additional file 1: Table S1.** Endorsed items on the stigma questionnaire in individuals meeting case criteria for likely PTSD, alcohol misuse and common mental disorders.** Table S2.** Stigma scale summary scores of those who do and do not meet case criteria for likely PTSD, alcohol misuse and common mental disorders.

## Data Availability

No additional data are available.

## Abbreviations

AF: Armed Forces
CMD: Common Mental Disorders
PTSD: Posttraumatic stress disorder
