# Supplementary material for: Perceived stigma and barriers to care in UK Armed Forces personnel and veterans with and without probable mental disorders
Source: BMC Psychol. 2019 Nov 27;7:75. doi: 10.1186/s40359-019-0351-7 (PMC6881983; doi:10.1186/s40359-019-0351-7)
Supplement: Supplementary file 1 — Additional file 1: Table S1. Endorsed items on the stigma questionnaire in individuals meeting case criteria for likely PTSD, alcohol misuse and common mental disorders. Table S2. Stigma scale summary scores of those who do and do not meet case criteria for likely PTSD, alcohol misuse and common mental disorders. [file 40359_2019_351_MOESM1_ESM.docx]

Supplementary Table 1

*Endorsed items on the stigma questionnaire in individuals meeting case criteria for likely PTSD, alcohol misuse and common mental disorders.*

|  | PTSD | | | Alcohol misuse | | | CMD | | |
| --- | --- | --- | --- | --- | --- | --- | --- | --- | --- |
| Stigma items | AOR (95% CI) | % agree non-cases | % agree cases | AOR (95% CI) | % agree non-cases | % agree cases | AOR (95% CI) | % agree non-cases | % agree cases |
| *Access to mental health services* |  | | | | | | | | |
| It’s difficult to schedule an appointment | **3.36 (2.13; 5.31)** | 12.5% | 32.5% | 1.03 (0.66; 1.60) | 14.3% | 14.4% | **2.63 (1.83; 3.80)** | 11.6% | 25.9% |
| It would be difficult to get time off work for treatment | **2.22 (1.44; 3.42)** | 15.8% | 29.2% | 1.36 (0.94; 1.96) | 16.3% | 20.2% | **2.03 (1.46; 2.83)** | 14.7% | 26.7% |
| I don’t know where to get help | 1.33 (0.64; 2.71) | 6.3% | 8.2% | 0.99 (0.56; 1.74) | 6.4% | 6.7% | 1.25 (0.74; 2.10) | 6.1% | 7.8% |
| I don’t have adequate transport | 3.70 (0.96; 14.20) | 0.7% | 2.5% | 1.45 (0.39; 5.45) | 0.8% | 1.2% | 2.80 (0.86; 9.06) | 0.6% | 1.9% |
| *Internalised stigma of mental illness* |  | | | | | | | | |
| Members of my unit might have less confidence in me | **2.43 (1.61; 3.65)** | 41.1% | 62.9% | **1.48 (1.10; 1.98)** | 41.3% | 50.6% | **1.49 (1.13; 2.00)** | 40.9% | 51.6% |
| My unit bosses might treat me differently | **3.35 (2.17; 5.19)** | 46.0% | 73.8% | **1.33 (1.03; 1.79)** | 47.3% | 54.0% | **1.94 (1.45; 2.58)** | 45.3% | 61.9% |
| It would harm my career | **2.35 (1.56; 3.50)** | 36.5% | 56.7% | **1.49 (1.11; 2.00)** | 36.4% | 46.0% | **1.68 (1.26; 2.24)** | 35.7% | 49.0% |
| I would be seen as weak by those who are important to me | **4.67 (2.99; 7.29)** | 37.1% | 73.1% | **1.74 (1.30; 2.34)** | 37.8% | 51.3% | **2.26 (1.69; 3.02)** | 36.4% | 56.9% |
| It would be too embarrassing | **2.48 (1.68; 3.71)** | 32.8% | 54.6% | **1.79 (1.34; 2.41)** | 32.2% | 46.7% | **1.78 (1.34; 2.35)** | 32.1% | 45.9% |
| My bosses would blame me for the problem | **5.38 (3.44; 8.42)** | 14.2% | 47.1% | **1.59 (1.09; 2.32)** | 15.6% | 22.1% | **3.31 (2.35; 4.66)** | 13.1% | 33.6% |
| Concern about what my friends or family might think | **3.13 (2.10; 4.66)** | 30.4% | 57.8% | **1.94 (1.45; 2.60)** | 29.9% | 45.4% | **2.17 (1.63; 2.87)** | 29.2% | 47.5% |
| *Perceived stigma of mental health care/providers* |  | | | | | | | | |
| My visit would not remain confidential | **2.38 (1.43; 3.96)** | 9.7% | 20.4% | **1.81 (1.19; 2.76)** | 9.6% | 15.4% | **1.94 (1.30; 2.90)** | 9.2% | 16.7% |
| I would think less of a team member or colleague if I knew they were receiving mental health counselling | 1.41 (0.68; 2.91) | 6.0% | 8.2% | 1.51 (0.89; 2.55) | 5.7% | 8.4% | 1.09 (0.62; 1.93) | 6.1% | 6.6% |
| I’ve had bad experiences with mental health professionals | **4.29 (2.76; 6.66)** | 10.6% | 33.7% | 1.49 (.99; 2.24) | 11.9% | 16.3% | **2.70 (1.88; 3.90)** | 10.1% | 23.5% |
| I don’t trust mental health professionals | **3.71 (2.04; 6.74)** | 5.7% | 18.1% | 1.57 (0.92; 2.66) | 6.2% | 9.0% | **2.52 (1.54; 4.12)** | 5.4% | 12.3% |
| My bosses discourage the use of mental health services | **4.73 (2.34; 9.54)** | 3.3% | 13.4% | 1.17 (0.55; 2.50) | 3.9% | 4.3% | **3.06 (1.68; 5.57)** | 3.0% | 8.8% |
| Mental health care doesn’t work | **4.06 (2.24; 7.38)** | 5.9% | 20.1% | **1.88 (1.10; 3.21)** | 6.1% | 11.0% | **2.32 (1.37; 3.89)** | 5.9% | 12.4% |
| Not wanting a mental health problem to be on my medical records | **2.89 (1.89; 4.44)** | 45.0% | 69.7% | **1.57 (1.17; 2.10)** | 45.3% | 55.3% | **1.80 (1.35; 2.40)** | 44.2% | 59.3% |
| Wanting to solve the problem on my own | **1.80 (1.15; 2.82)** | 60.8% | 73.6% | **2.20 (1.58; 3.07)** | 58.9% | 75.3% | **1.53 (1.13; 2.08)** | 60.0% | 70.0% |
| Mental health treatment has harmful side effects | **3.28 (1.83; 5.91)** | 7.7% | 21.6% | 1.03 (0.58; 1.83) | 8.6% | 9.3% | **2.74 (1.71; 4.40)** | 7.0% | 17.1% |

*Note*. CMD = common mental disorders, includes participants meeting case criteria on both the GAD and PHQ. AOR = adjusted odds ratio. Adjusted for sex and age. Results for those who responded ‘neither agree nor disagree’ are not presented. * indicates a significant difference between mean scores at p>0.05

Supplementary Table 2

*Stigma scale summary scores of those who do and do not meet case criteria for likely PTSD, alcohol misuse and common mental disorders.*

| Stigma scales |  | PTSD | Alcohol misuse | CMD |
| --- | --- | --- | --- | --- |
| *Access sub-scale summary score, M(SD)* | Case M(SD) [95% CI] | 0.61 (0.89)* [0.42; 0.80] | 0.36 (0.63) [0.27; 0.46] | 0.48 (0.77)* [0.37; 0.59] |
|  | Non-case M(SD) [95% CI] | 0.28 (0.60) [0.25; 0.33] | 0.30 (0.63) [0.26; 0.35] | 0.27 (0.59) [0.23; 0.31] |
| *Internalised stigma sub-scale summary score, M(SD)* | Case M(SD) [95% CI] | 3.95 (2.68)* [3.24; 4.66] | 2.69 (2.81)* [2.20; 3.19] | 3.09 (2.83)* [2.61;3.59] |
|  | Non-case M(SD) [95% CI] | 1.90 (2.48) [1.71; 2.09] | 1.93 (2.48) [1.73; 2.13] | 1.83 (2.43) [1.63;2.03] |
| *Perceived stigma of mental health care/providers sub-scale summary score* | Case M(SD) [95% CI] | 2.17 (1.89)* [1.52; 2.82] | 1.29 (1.38) [0.97; 1.62] | 1.52 (1.60)* [1.16; 1.88] |
|  | Non-case M(SD) [95% CI] | 0.99 (1.21) [0.88; 1.09] | 1.03 (1.28) [0.91; 1.15] | 0.99 (1.22) [0.87; 1.10] |

*Note*. CMD = common mental disorders, includes participants meeting case criteria on both the GAD and PHQ. CI = confidence interval. * indicates a significant difference between mean scores at p>0.05
